# Cross brain reshaping in congenital visual or hearing impairment: triple-network dysfunction

**DOI:** 10.1093/braincomms/fcaf150

**Published:** 2025-04-17

**Authors:** Jiahong Li, Binbin Xiong, Suijun Chen, Jing Li, Yingting Luo, Yu-Chen Chen, Jae-jin Song, Fei Zhao, Jing Yang, Chenlong Li, Yiqing Zheng, Lan Gui, Huanling Feng, Weirong Chen, Yuexin Cai, Wan Chen

**Affiliations:** Department of Otolaryngology, Sun Yat-sen Memorial Hospital, Sun Yat-sen University, Guangzhou, Guangdong 510120, China; Institute of Hearing and Speech-Language Science, Sun Yat-sen University, Guangzhou City, Guangdong Province 510120, China; Center for Hearing and Balance, Zhuhai Hospital of Integrated of Traditional Chinese Medicine and Western Medicine, Zhuhai, Guangdong 519000, China; Department of Otolaryngology, Sun Yat-sen Memorial Hospital, Sun Yat-sen University, Guangzhou, Guangdong 510120, China; Institute of Hearing and Speech-Language Science, Sun Yat-sen University, Guangzhou City, Guangdong Province 510120, China; Department of Otolaryngology, Sun Yat-sen Memorial Hospital, Sun Yat-sen University, Guangzhou, Guangdong 510120, China; Institute of Hearing and Speech-Language Science, Sun Yat-sen University, Guangzhou City, Guangdong Province 510120, China; Zhongshan school of medicine, Sun Yat-sen University, Guangzhou, Guangdong 510080, China; Department of Radiology, Nanjing First Hospital, Nanjing Medical University, Nanjing 210012, China; Department of Otorhinolaryngology, Seoul National University Bundang Hospital, Seongnam-si 03080, South Korea; Department of Speech and Language Therapy and Hearing Science, Cardiff Metropolitan University, Cardiff CF5 2YB, United Kingdom; Center for Hearing and Balance, Zhuhai Hospital of Integrated of Traditional Chinese Medicine and Western Medicine, Zhuhai, Guangdong 519000, China; Weldon School of Biomedical Engineering, Purdue University, West Lafayette, IN 47907, USA; Department of Otolaryngology, Sun Yat-sen Memorial Hospital, Sun Yat-sen University, Guangzhou, Guangdong 510120, China; Institute of Hearing and Speech-Language Science, Sun Yat-sen University, Guangzhou City, Guangdong Province 510120, China; Department of Otolaryngology, Shenshan Medical Center, Memorial Hospital of Sun Yat-sen University, Guangzhou, Guangdong 516621, China; Department of Otolaryngology, Shenshan Medical Center, Memorial Hospital of Sun Yat-sen University, Guangzhou, Guangdong 516621, China; State Key Laboratory of Ophthalmology, Zhongshan Ophthalmic Center, Sun Yat-sen University, Guangzhou, Guangdong 510060, China; State Key Laboratory of Ophthalmology, Zhongshan Ophthalmic Center, Sun Yat-sen University, Guangzhou, Guangdong 510060, China; Department of Otolaryngology, Sun Yat-sen Memorial Hospital, Sun Yat-sen University, Guangzhou, Guangdong 510120, China; Institute of Hearing and Speech-Language Science, Sun Yat-sen University, Guangzhou City, Guangdong Province 510120, China; State Key Laboratory of Ophthalmology, Zhongshan Ophthalmic Center, Sun Yat-sen University, Guangzhou, Guangdong 510060, China; Hainan Eye Hospital and Key Laboratory of Ophthalmology, Zhongshan Ophthalmic Center, Sun Yat-sen University, Haikou, Hainan 570311, China

**Keywords:** congenital visual impairment, congenital hearing impairment, functional connectivity, resting-state electroencephalography, support vector machine

## Abstract

This research examines how congenital visual or hearing impairment reshapes brain function using EEG. The study involved 40 children with congenital visual impairment, 40 with hearing impairment and 42 age and gender-matched normal children as controls. The investigation included assessments of visual and auditory abilities, along with comprehensive EEG evaluations. Techniques such as source localization, functional connectivity and cross-frequency coupling were used to analyse variations in brain activity. Machine learning methods, specifically support vector machines, were utilized to identify key reshaping characteristics associated with congenital impairments. Results showed reduced activation in the visual cortex for visually impaired children and decreased activation in the auditory cortex for hearing-impaired children compared with the control group. Both impairment groups demonstrated significant reductions in functional connectivity across various brain regions, including the visual and auditory cortices, insula, parahippocampal gyrus, posterior cingulate gyrus and frontal cortex. The machine learning model highlighted aberrant connectivity between the visual/auditory cortex and the right insula, the medial prefrontal cortex and dorsolateral prefrontal cortex and the visual and auditory cortex in children with these impairments in the alpha frequency band. Spatially similar patterns of cross-frequency coupling of rhythmic activity were also observed. The study concludes that congenital visual and hearing impairments significantly impact brain development, identifying distinct functional characteristics and shared reshaping patterns. The consistent presence of dysrhythmic activity and reduced functional connectivity suggest the existence of a triple network anomaly.

## Teaser

Li *et al*. report that the children with congenital visual and hearing impairments result in decreased sensory cortex activation, reduced functional connectivity and altered cross-frequency coupling patterns across brain regions, highlighting a Triple-Network dysfunction through EEG and machine learning analysis.

## Graphical Abstract

**Graphical Abstract:**
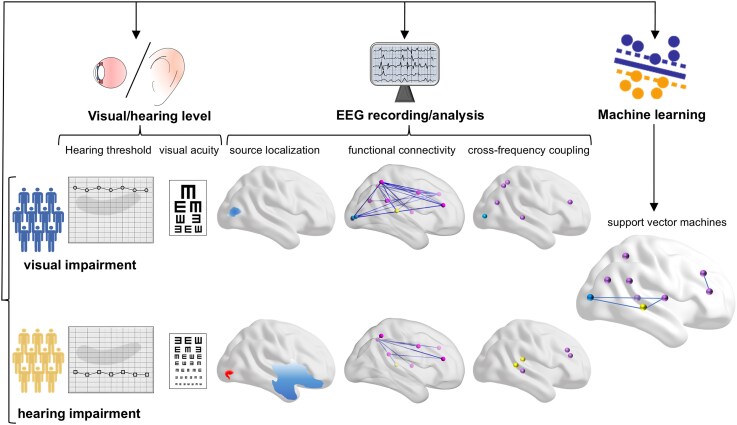

## Introduction

Normal visual and auditory functions serve as essential cornerstones for the healthy development of the brain and the acquisition of knowledge and experiences. Additionally, they play pivotal roles in the growth of advanced cognitive functions, including memory, attention, emotions and executive functions, particularly during the school-age years.^1^ On one hand, the formation of human behavioural capabilities relies on the integrity of sensory functions, facilitating efficient information intake, knowledge acquisition and the accumulation of life experiences. On the other hand, sensory input information is projected through a bottom-up transmission pathway to the central sensory cortex, thereby fostering the development of higher-level central functions.

Congenital deprivation of peripheral sensory functions has been demonstrated to lead to varying degrees of cognitive, behavioural or brain functional changes, as supported by research evidence in the fields of behavioural science and neuroimaging.^2^ For instance, a meta-analysis has indicated that the impact of hearing loss on brain alterations is diverse. Congenital hearing loss affects the structural integrity of various brain regions, demonstrating dynamic adaptation and compensation. General characteristic changes are not limited to the temporal cortex but also affect the visual system, a significant decline in gray matter volume in the frontal cortex, and other adaptations.^3^ Similarly, congenital visual impairment during critical developmental periods can induce significant plastic changes in intact brain regions, including the cingulate gyrus, insula, temporal cortex and frontal cortex.^4^ This suggests that congenital sensory deficits in vision and hearing manifest complex neuroplasticity in central brain structures and functions, extending beyond sensory cortices and involving pathological alterations across cortical areas.

Furthermore, children with sensory deprivation in different modalities exhibit similar cross-modal plasticity features. A typical example is that congenitally deaf and blind children both demonstrate enhanced coupling between the visual and auditory systems, along with changes in cortical areas involved in behavioural aspects such as executive functions and attention control.^5^ This suggests the brain's capacity to adjust and reorganize its neural structures following sensory loss or impairment. Neuroimaging evidence indicates that these plasticity mechanisms are associated with widespread changes in brain network patterns, and early deficits in visual/auditory functions can negatively impact attention and other higher-order cognitive networks. For instance, Liu *et al.* found that congenitally blind individuals showed reduced functional connectivity within the occipital area and between the occipital cortex and parietal somatosensory, frontal motor and temporal multisensory cortices. However, introducing Braille and longer daily practice in early life led to stronger functional connectivity between these brain regions.^6^ By conducting a functional magnetic resonance imaging-based tactile perception task study, Ankeeta *et al.*^7^ observed increased functional connectivity in congenitally blind children between the visual and dorsal attention, somatosensory-motor networks and between the Default Mode Network (DMN) and Salience Network (SN) with functional outcomes correlating with the duration of sensory training. In another study using functional magnetic resonance imaging and behavioural tasks such as the Token Test, children with congenital single-sided deafness exhibited reduced connectivity in the DMN and fronto-parietal network, decreased activation in the anterior and posterior brain regions of the DMN, suggesting developmental features of brain networks related to cross-modal sensory processing.^8^ In children with congenital sensory deficits, multiple functional connectivity differences were found in brain networks associated with executive functions, cognition and language comprehension, which may represent adaptive and maladaptive changes. Early interventions for such sensory deficits with sign language or hearing aids normalize connectivity between the primary sensory cortex and attention networks.^9^ These commonalities in brain network remodeling highlight the universal impact of sensory deprivation on brain network patterns, with a focus not only on the visual- and auditory networks but also on the DMN, SN and fronto-parietal network. Importantly, the significance of network pattern changes underscores the development of structural and functional connectivity in core neurocognitive brain networks as crucial for the maturation of increasingly complex cognitive abilities. For example, in early life, if a child lacks visual input, sensory patterns are typically not considered competing factors for neural resources. Thus, even in cases of cataract reversal, these children with cataracts may still perform poorly in tasks related to global motion perception (detecting the overall movement of visual stimuli) compared with children with normal vision.^10^ This implies that the timing of interventions following sensory deprivation is crucial for inducing neural plasticity.

Utilizing the theoretical framework of the brain network model, we can delve deeper into unravelling the intricacies and interconnections involved in central functional reshaping in cases of congenital sensory deprivation. Given the anomalous core brain network systems associated with congenital visual/hearing impairment, the triple-network model provides a new perspective for understanding the mechanisms and clinical manifestations of these conditions.^11,12^ Compared with other theoretical framework, the Triple-Network Model better reveals the link between congenital sensory loss and abnormal brain network functioning. This model was introduced by Menon *et al.* in 2010 and consists of three core neurocognitive networks involved in higher-order attention and cognitive control processes: SN, Central Executive Network (CEN) and DMN.^13^ The model posits that the maturation of cognitive abilities such as working memory, attention and cognitive control is achieved through interactions within and between these three large-scale brain networks. The DMN is typically active during rest or when not focused on specific tasks. It primarily engages in activities related to introspection, self-reflection, emotion processing and personal thoughts, playing a crucial role in non-task-specific thinking, memory recall, self-awareness and social cognition. The CEN is a brain network associated with cognitive control and task-oriented thinking. It coordinates the collaboration of different brain regions to meet specific goals and task requirements. The SN plays a key role in perceptual-attention switching and emotional regulation. It modulates processes such as attention allocation, emotional experience and decision-making, mediating interactions between large brain networks involved in externally oriented attention and internally directed cognition. Early evidence suggests that children with sensory deprivation exhibit abnormal connectivity patterns across multiple networks, and this can lead to behavioural dysregulation and central changes at various levels.^14^ However, it is currently unclear whether children with congenital sensory deprivation display common manifestations of disruption in the triple network. Therefore, further exploration of the mechanistic patterns of central remodeling caused by visual and auditory deficits is needed.

Brain rhythms reflect patterns of neural activity across different frequency ranges, revealing the brain's functional states and dynamic changes in cognition, emotional regulation and sensory processing. The alpha frequency band (typically 8–13 Hz) is one of the most prominent brain wave types during resting states and plays a significant role in modulating responses to external stimuli. Extensive research has demonstrated that alpha activity is closely associated with the selective inhibition of sensory information.^15^ Specifically, increased alpha activity is often interpreted as a mechanism of inhibition, reducing the impact of irrelevant or distracting sensory input, thereby optimizing the processing of relevant information.^16^ For example, in visual tasks, an increase in alpha activity in non-task-related regions is thought to help suppress sensory channels (such as auditory input) unrelated to the current task, thus enhancing the efficiency of processing task-relevant information.^17^ Furthermore, the alpha band is implicated in broader neural regulatory functions, particularly in attention allocation.^18^ Research has shown that changes in alpha wave synchronization are associated with performance on attention tasks, suggesting that alpha oscillations may play a role in managing interference and optimizing cognitive resource utilization.^19,20^ Consequently, changes in alpha activity may reflect neural compensatory mechanisms during the processing of external stimuli, influencing the integration of cognitive and sensory functions. Exploring brain rhythms, particularly changes in the alpha band, aids in understanding its adaptive regulatory role in neural network reorganization under conditions of reduced sensory input.

In this study, we recruited children with congenital cataracts and congenital middle and external ear deformities as participants to represent individuals with congenital visual /hearing impairments. Using electroencephalography (EEG), we measured their resting-state cortical activity and employed analytical techniques such as source localization, functional connectivity and cross-frequency coupling. Additionally, we have incorporated machine learning techniques, specifically support vector machine (SVM) methods, to uncover shared or similar central neuroplasticity features among these participants. This research aims to help us explore the central nervous system functional changes resulting from congenital visual /hearing impairments. Although previous research has explored the impact of congenital sensory deficits on brain structure and certain functional networks, there is still a lack of systematic studies on how these deficits lead to functional reorganization across multiple core neural networks. Moreover, many existing studies have primarily focused on the effects of single sensory deficits. In contrast, this study combines visual and auditory deficits to compare their similarities and differences. By identifying the specific patterns of these changes, we aim to gain a better understanding of the long-term effects of sensory loss on brain structure and function. This will contribute to a more comprehensive understanding of the pathological mechanisms induced by sensory deficits and provide insights for clinical interventions and rehabilitation. By examining the specific networks involved in abnormal functional connectivity, we can develop more targeted therapeutic approaches with the goal of restoring normal brain function and cognitive development in affected children. Finally, the results of this study not only contribute to the existing foundational neuroscience research but also advance the exploration of the impacts of other types of sensory deficits, thereby providing new research directions in the field of neurodevelopment. We believe that these additional insights will more comprehensively highlight the significance and forward-looking nature of this research and will have a positive impact on future scientific investigations and clinical practice.

## Materials and methods

### Participants

A total of forty children with congenital (present at birth or before the age of one) cataracts and forty children with congenital middle and external ear deformities were recruited from the Ophthalmology Center of Sun Yat-sen University and the Sun Yat-sen Memorial Hospital's Department of Otorhinolaryngology. Additionally, 42 age and sex-matched children without visual- or auditory problems were recruited as a control group. Informed written consent was obtained from the legal guardians of all participating children in this study. The research protocol was approved by the Ethics Committee of the Sun Yat-sen Memorial Hospital (Ethics Number: SYSKY-2023-1000-01), and the Ophthalmology Center of Sun Yat-sen University (Ethics Number: 2023KYPJ309). None of the enrolled children had received interventions such as hearing aids, vision aids (such as corrective glasses) or surgical procedures (e.g. bone-anchored hearing aids, cochlear implants or intraocular lens implants) and reported the presence of tinnitus.

For children with congenital cataracts, all patients had unilateral or bilateral cataracts classified as Grade III according to the Lens Opacities Classification System (LOCS III), with corneal astigmatism within 1.0 diopters (D). Exclusion criteria included a history of neurological or psychiatric diseases, prior refractive errors, glaucoma or penetrating keratoplasty, degenerative ocular conditions, and any ocular or systemic conditions that could potentially affect the study outcomes. However, the children with congenital middle- and external ear deformities presented with conductive hearing loss due to either uni- or bilateral congenital aural atresia or abnormal development of the middle and external ear. Exclusion criteria included non-congenital ear deformities or ear abnormalities with other known aetiologies, a history of neurological or psychiatric diseases, prior surgical interventions, systemic illnesses or recent use of hearing aids or other audiological interventions. All patients have completed dual-eye visual acuity assessment [using Snellen visual acuity charts, and the visual acuity data were recorded for the right eye (OD) and the left eye (OS), respectively] and bilateral hearing threshold evaluation (using pure-tone audiometry, and pure tone averages were calculated by taking the average of the thresholds at 0.5 kHz, 1 kHz, 2 kHz, 4 kHz and 8 kHz for the left and right ear, respectively), with the degree of visual impairment or hearing loss on the weaker side defined as the maximum deficit. All details are shown in Table 1.

**Table 1:** Participants characteristics

	Congenital visual impairment (*n* = 40)	Congenital hearing impairment (n = 40)	Healthy control (*n* = 42)	F/X^2^, *P*
**Age** (y) (mean ± SD)	7.92 ± 2.28	8.32 ± 1.45	8.83 ± 2.21	F = 2.103, *P* = 0.127
**Gender** (male: female)	25:15	28:12	23:19	X^2^ = 2.026, *P* = 0.363
**Laterality** (left: right: bilateral)	10:7:23	13:14:13	——	X^2^ = 5.502, *P* = 0.064
**Maximum hearing loss** (dB HL)	<20	70.30 ± 7.43	<20	——
**Maximum apparent loss** (OD/OS)	0.30 ± 0.19	>1.0	>1.0	——

Note: OD (Oculus Dexter): represents the right eye; OS (Oculus Sinister): represents the left eye; dB HL (decibels Hearing Level) represents the threshold of hearing.

### Recording and preprocessing of resting-state EEG

The EGI's 128-channel high-density saline EEG to collect resting-state EEG signals from all subjects. The software version used in the acquisition process is Netstation 4.3 and is paired with an AMP 300 amplifier. The following are the parameter settings for acquisition: the original sampling rate is 1000 Hz/s, the bandpass filtering range is set to 0.1∼200 Hz, the Cz electrode is used as the original reference electrode and the impedance is controlled below 50 KΩ. All EEG measurements were carried out in an electromagnetically shielded room, and subjects were asked to sit in a comfortable chair with their eyes closed and minimize head and body movements to maintain mental relaxation. The duration of the entire data collection process is approximately 7 min.

After EEG data collection, offline processing was carried out using MATLAB vR2018b and EEGLAB v2020 toolboxes. The key steps of pre-processing include the following aspects: first, the sampling rate of the data is reduced to 500 Hz/s, and 50 Hz dip filtering is performed to remove power frequency interference, and 1–50 Hz bandpass filtering. Then, a large range of drifting fragments were manually removed and the damaged electrodes were replaced by interpolation. Then, the possible physiological artefacts such as blinking, eye movement and head movement were eliminated by independent component analysis. For frequency domain analysis, data is divided into different frequency bands: Delta (1–4 Hz), Theta (4–8 Hz), Alpha (8–13 Hz), Beta (13–30 Hz) and Gamma (30–44 Hz).^21,22^ The data for each frequency band is segmented into 2-second segments and converted to txt format for subsequent analysis. To ensure data quality, we referred to methods from previous EEG studies and applied *Z*-score transformation to detect potential outliers. Data points with *Z*-scores exceeding ±5 standard deviations were considered outliers and excluded from subsequent machine learning analysis.^23^ This step aimed to eliminate extreme values to prevent potential distortion of the results.

### EEG source localization

sLORETA (Standardized Low Resolution Electromagnetic Brain Tomography, developed by a research team from the University of Lausanne in Switzerland, led by Dr. Roberto D. Pascual-Marqui, available at http://www.uzh.ch/keyinst/loreta.htm) is a software used to estimate the location of intracranial power sources in EEG electrical signals recorded by the scalp, revealing patterns of activity in brain regions by calculating the current density of electrical activity in neurons. The the current density (in A/m^2^) of the electrical activity of the neurons is calculated without specifying the number of signal sources in advance.^24^ sLORETA uses a standardized three-layer spherical brain model, including cortex, gray matter and white matter, to establish spatial localization of electromagnetic sources. To solve the inverse problem, which is to inversely deduce the brain power source position and current density from scalp potential data, sLORETA adopts a regularization method. Moreover, a mathematical model was established between scalp potential data and voxels in the source space by the least squares estimation method, and the current density distribution was estimated by minimizing the error between the measured data and the model.^25^ The sLORETA source space is composed of 6239 × 5 voxels, each of which is 5 × 5 mm in size, and is defined by MNI (Montreal Neurological Institute) coordinate.^26^ sLORETA toolbox has undergone several validation studies, including functional magnetic resonance imaging, structural magnetic resonance and PET localization techniques. These studies have endorsed sLORETA's reliability and effectiveness in determining the source location of EEG signals.^27^

### Functional connectivity analysis

To explore the correlation between regions, we calculated functional connectivity coefficients to measure the functional connections of brain regions. Synchronization between time series corresponding to different spatial locations is often used as an indicator to evaluate “functional connectivity”. Relevant functional connectivity parameters, including the lagged coherence parameter, can be calculated during functional connectivity analysis of a specified brain functional area.^28^ Lagged coherence introduced the concept of time delay in addition to coherence. It reflects the relationship between the frequency components of the signal in different time series, that is, the degree of synchronization of different signals in frequency.^29^ The value of lagged coherence ranges from 0 to 1, indicating the degree between no synchronization and complete synchronization. A higher coherence value indicates a strong synchronization relationship, while a lower coherence value indicates a lack of synchronization or greater randomness. This measure of dependence can be applied jointly to any number of brain regions, including functional regions distributed in cortical networks. By calculating the lagged coherence parameter, the synchronization between different brain regions can be quantified, revealing the pattern and strength of functional connections. The application of this method is not limited by the number of brain regions; so, complex cortical networks can be analysed and the functional connectivity of the brain can be studied in depth. By using the lagged coherence parameter, we were able to gain important insights into information transfer and coordination between different brain regions.

Its calculation formula is as follows:


$$\mathrm{lagged}\;coherence=\sqrt{\frac{Im{\left[S_{xy}(\omega )\right]}^{2}}{S_{xx}(\omega )S_{yy}(\omega )-Re{\left[S_{xx}(\omega )\right]}^{2}}}$$

Where *Im* and *Re* represent the imaginary and real parts of the Fourier transform respectively; S_xy_(ω) is the cross-power spectrum of X(ω) and Y(ω); S_xx_(ω) and S_yy_(ω) are the self-power spectral densities of X(ω) and Y(ω).

According to the triple network theory and previous literature,^13,30^ the core areas of interest were selected as follows: **Visual Network (VN):** bilateral primary and secondary visual cortex (V1, V2); **Auditory Network (AN):** bilateral primary and secondary auditory cortex (A1, A2); **Default Mode Network (DMN):** bilateral parahippocampal gyrus (PHC), posterior cingulate cortex (PCC), angular gyrus (AG), medial prefrontal cortex (MPFC); **Salience Network (SN):** bilateral insula (INS), dorsal anterior cingulate cortex (dACC) and **Central Executive Network (CEN):** bilateral dorsolateral prefrontal cortex (DLPFC) and posterior parietal cortex (PPC). In this way, we explored a total of five network systems involving 24 regions of interest (ROIs; see Table 2).

**Table 2:** The detail of 24 region of interest

Network system	Name of the brain region	Brodmann areas	Abbreviation
Visual network (VN)	Primary visual cortex	BA17	V1
	Secondary visual cortex	BA18	V2
Auditory network (AN)	Primary auditory cortex	BA41	A1
	Secondary auditory cortex	BA42	A2
Default mode network (DMN)	Parahippocampal gyrus	BA27	PHC
	Posterior cingulate cortex	BA23	PCC
	Angular gyrus	BA39	AG
	Medial prefrontal cortex	BA46	MPFC
Salience network (SN)	Insula	BA13	INS
	Dorsolateral anterior cingulate gyrus	BA24	dACC
Central executive network (CEN)	Dorsolateral prefrontal cortex	BA09	DLPFC
	Posterior parietal cortex	BA40	PPC

### Machine learning–based classification

In order to further search for discernible brain network connection patterns, we combined SVM to find the common characteristics of network connection changes between the two study groups. The SVM is a commonly used supervised learning method for classification and regression analysis. It has a wide range of applications in the field of machine learning and has demonstrated its unique advantages in tasks such as pattern recognition, image processing and disease prediction.^31^ SVM achieves data classification by constructing the maximum margin hyperplane in the feature space, which has strong generalization ability and robustness. In binary classification problems, the goal of the SVM is to find a maximum margin hyperplane that maximizes the interval between classes (i.e. the maximum distance between two classes). The hyperplane with the greatest interval is called the optimal hyperplane. For nonlinearly separable data, SVM can separate the data linearly in the new space by using kernel functions to map features into a high-dimensional space.

In this study, SVM algorithm in data mining software WEKA (Waikato Environment for Knowledge Analysis version 3.8.6, developed by the University of Waikato Machine Learning Group, available at http://www.cs.waikato.ac.nz/ml/weka/) was used to perform the classification of data sets. The Weka software suite includes a library of algorithms that build predictive models by learning from user-provided data sets. We use the default settings as the running parameters. Our dataset included functional connection coefficients (pin-to-pin-brain connections, 276 coefficient values) for each subject in five frequency bands (i.e. delta, theta, alpha, beta and gamma) for a total of 24 ROIs and compared to normal controls. The criterion for correct classification is to assign subjects to the correct group based on the model calculated by the WEKA software (for example, for the complete model: disease group versus health group). We used 10-fold cross-validation to evaluate the performance of the SVM classifier in WEKA v3.8.6. Ten-fold cross-validation is a common model evaluation method that assesses the performance and generalization ability of classifiers. This method divides the dataset into ten equal-sized subsets, with nine subsets used as training data and the remaining one as test data, repeating this process ten times. By comprehensively evaluating the results from each iteration, we can derive a reliable performance evaluation index for the classifier and draw robust conclusions.

### Cross-frequency coupling analysis

In order to deeply reveal the mechanism of abnormal brain network pattern of decreased audiovisual function, cross-frequency coupling analysis can be used to explore the spatial distribution of rhythm disturbance in different brain regions of sensory deprivation. Cross-frequency coupling analysis is a method used to study the coupling relationship between different frequencies in EEG signals, and the degree of coupling is evaluated by calculating the correlation between the phase of the low-frequency signal and the amplitude of the high-frequency signal.^32^ Typically, the calculation can be performed using the phase-amplitude coupling method. The cross-frequency coupling Index (MI) is a commonly used index of phase-amplitude coupling analysis to describe the nonlinear coupling between different frequencies in EEG signals. MI algorithm, proposed by Tort *et al*.,^33^ is mainly based on the distribution of the amplitude of the high-frequency rhythm on the phase signal of the low-frequency rhythm to calculate the Shannon entropy of the distribution so as to characterize the non-uniformity of the distribution, and the maximum Shannon entropy in the uniform distribution is used for normalization. The low frequency (theta: 4–7 Hz) and high frequency (gamma: 30–45 Hz) are defined, that is, the coupling results of theta-gamma are calculated. Firstly, the phase signal θ_low_(t) of the low frequency rhythm and the amplitude signal A High(t) of the high frequency rhythm are extracted. Then, each period of phase signal θ_low_(t) is divided into *n* equal parts on average, and the mean value of the high frequency amplitude corresponding to the j phase interval < A _High_(t) > θ(j) is calculated. Then, the mean value of the corresponding amplitude of each phase interval is divided by the sum of the n means to obtain the distribution P(j) of the amplitude of the high frequency rhythm on the phase of the low frequency rhythm. The Shannon entropy H(P) of the amplitude distribution in phase is calculated based on P(j). When P(j) is uniformly distributed, H(P) will reach the maximum lb(n), so the above Shannon entropy can be normalized to MI:


$$MI=\frac{lb(n)-H(P)}{lb(n)},\;MI\in [0,1]$$

Therefore, MI measures the degree of coupling between different frequencies by quantifying the degree to which the phase of the low frequency oscillation modulates the amplitude of the high frequency oscillation. The larger the MI value, the stronger the modulation of high-frequency amplitude by low-frequency phase, indicating a stronger coupling between the low-frequency phase and the high-frequency amplitude. MI can be used to explore nonlinear interactions between frequencies in brain networks, such as how low frequency oscillations modulate high frequency oscillations to achieve functional coordination.

### Statistical analysis

In this study, we used SPSS 22.0 software to compare basic information (such as gender, age, etc.), used ANOVA and Chi-square test, and controlled the significance level (α) below 0.05 to determine whether there was a significant difference between the study group and the control group. In order to further identify the differences in brain region activation and functional connectivity between the disease group and the normal group, non-parametric permutation *T*-test in sLORETA software was used for statistical analysis.^34^ It is noteworthy that the non-parametric nature of sLORETA method enables it to perform statistical non-parametric mapping (SnPM) analysis without relying on any Gaussian hypothesis, thus improving the reliability and robustness of the results. We conducted *T*-test comparative analysis on the two groups of data, and carried out replacement test correction at the significance level (*P* < 0.05). The threshold of significance difference was obtained by conducting 5000 random permutation tests to ensure statistical significance of the results. In order to reduce the error caused by multiple comparisons, we retained only the voxels with a significance level of *P* < 0.05. For the comparison of cross-frequency coupling coefficients, we used the independent sample t test for comparison, and obtained the final significance result based on the false-positive discovery rate correction, with the significance level (α) controlled at 0.05.

## Results

### Source-localized cortical activity differences between groups

**Figure 1:**
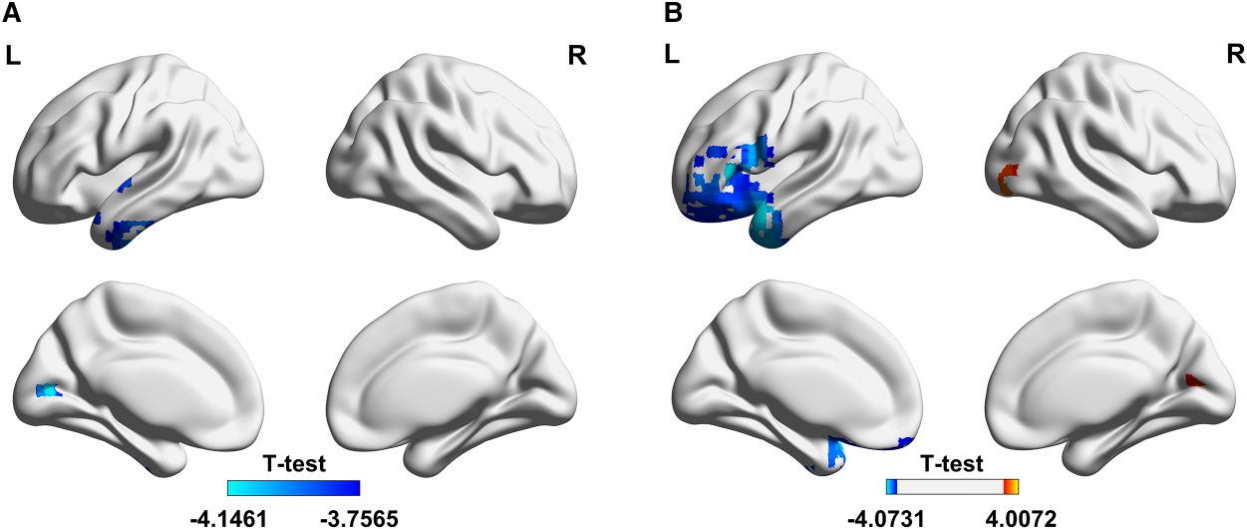
**Resting-state cortical activity differentials in congenital sensory impairments compared with the normal group.** To investigate the remodeling of cerebral cortices caused by visual and hearing impairments, sLORETA was applied to localize intracranial power sources, utilizing the statistical method of random permutation *t*-tests (the significance level alpha is <0.05). (**A**) In children with congenital visual impairment, a significant reduction in the left occipital cortex (BA 23) in alpha frequency band activity compared to the normal group (*t* < −3.75, df = 80, *P* < 0.05). (**B**) In children with congenital hearing impairment, a significant reduction in cortical activity in the left superior temporal gyrus (BA 38) and the left inferior frontal gyrus (BA 47) within the alpha frequency band (*t* < −4.07, df = 80, *P* < 0.05) compared with the normal group. Additionally, children with congenital hearing impairment exhibited increased activity in the right middle occipital cortex (BA 18) in the beta frequency band compared to the normal group (*t* > 4.01, df = 80, *P* < 0.05). The colour map shows the magnitude of the difference in current density, which is the *t* value of the nonparametric permutation T test calculated by sLORETA. L, left; R, right.

We first used inter-group comparison to analyse the features of remodeling of the cerebral cortices caused by visual/hearing impairment. Under sLORETA's random permutation *t-*test, we found that compared with the normal group, the resting-state cortical activity of the left occipital cortex (BA 23) in children with congenital visual impairment (*t* < −3.75, df = 80, *P* < 0.05) was reduced in the alpha frequency band. Meanwhile, compared with normal group for children with congenital hearing impairment, the resting-state cortical activity of the left superior temporal gyrus (BA38) and the left inferior frontal gyrus (BA47) ( < −4.07, df = 80, *P* < 0.05) is weakened in the alpha frequency band. However, the cortical activity of the right middle occipital cortex (BA 18) (*t* > 4.01, df = 80, *P* < 0.05) was enhanced for the beta frequency band (see Fig. 1). No significant differences were found in other frequency bands and brain regions.

### Functional connectivity analysis

**Figure 2:**
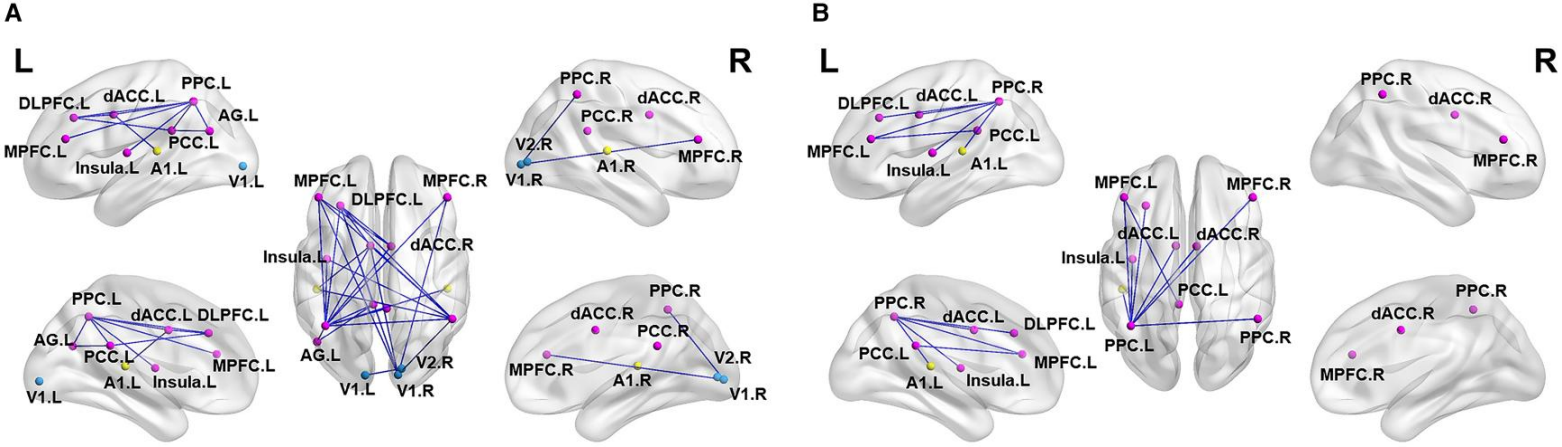
**Functional connectivity map of congenital sensory impairments compared to a normal group.** To explore the remodeling of functional connectivity due to visual and hearing impairments, sLORETA was used to calculate hysteresis coherence as an indicator of the functional connectivity of brain networks using statistical methods of a random permutation *t*-test (the significance level alpha is <0.05). (**A**) In children with congenital visual impairment, there is a widespread reduction in functional connectivity, including the V1, INS, dACC, MPFC, AG, PCC, DLPFC and PPC, compared with the normal group (*t* < −3.93, df = 80, *P* < 0.05). (**B**) In children with congenital hearing impairment, there is a significant reduction in functional connectivity, including the A1, INS, dACC, MPFC, AG, PCC, DLPFC and PPC, compared with the normal group (*t* < −3.74, df = 80, *P* < 0.05). Blue dots represent the visual network, yellow dots represent the auditory network and purple dots denote the triple-network (including the default mode network, salience network and central executive network). Blue lines indicate statistically significant reductions in functional connectivity compared to the normal group. L, left; R, right.

sLORETA-based lagged coherence functional connectivity coefficient analysis, with statistical evaluation using random permutation *t*-tests, found that both congenital hearing impairments and congenital visual impairments showed similar abnormal changes in brain network functional connectivity. However, these changes involved weakened functional connectivity across a wide range of brain regions, when compared to the normal control group, respectively. Specifically, the reduced functional connections in children with congenital visual impairment compared to the normal group involved brain regions including the V1, INS, dACC, MPFC, AG, PCC, DLPFC and PPC (*t* < −3.93, df = 80, *P* < 0.05). Similarly, in the group of children with congenital hearing impairment, weakened functional connections involved brain regions such as the A1, INS, dACC, MPFC, AG, PCC, DLPFC and PPC (*t* < −3.74, df = 80, *P* < 0.05; see Fig. 2).

### Classification of functional connections by SVM model

To identify common or similar network connections between individuals with congenital visual impairment and those with congenital hearing impairment, we used functional connection coefficients as feature variables and applied SVM model for classification. Two independent models were constructed: one comparing congenital hearing impairments to the normal control group, and another comparing congenital visual impairments to the normal control group. Comparing the performance of these models across different frequency bands, we observed that the highest classification accuracy was achieved in the alpha frequency band. As in the model comparing congenital visual impairment with the normal group, the classification accuracy was 75.6% (sensitivity: 77.5%; specificity: 77.5%). However, in the model comparing congenital hearing impairment with the normal group, the classification accuracy was 79.3% (sensitivity: 79.1%; specificity: 77.5%). The performance metrics of SVM models for other frequency bands were as follows: for the congenital visual impairment model, delta (accuracy: 53.7%; sensitivity: 55%; specificity: 53.9%), theta (accuracy: 63.4%; sensitivity: 66.7%; specificity: 70%), beta (accuracy: 68.3%; sensitivity: 73.5%; specificity: 77.5%) and gamma frequency band (accuracy: 65.9%; sensitivity: 63.5%; specificity: 52.5%); for the congenital hearing impairment model, delta (accuracy: 53.7%; sensitivity: 54.8%; specificity: 52.5%), theta (accuracy: 69.5%; sensitivity: 71.8%; specificity: 72.5%), beta (accuracy: 74.4%; sensitivity: 75.6%; specificity: 75%) and gamma frequency band (accuracy: 56.1%; sensitivity: 55.6%; specificity: 40%).

**Figure 3:**
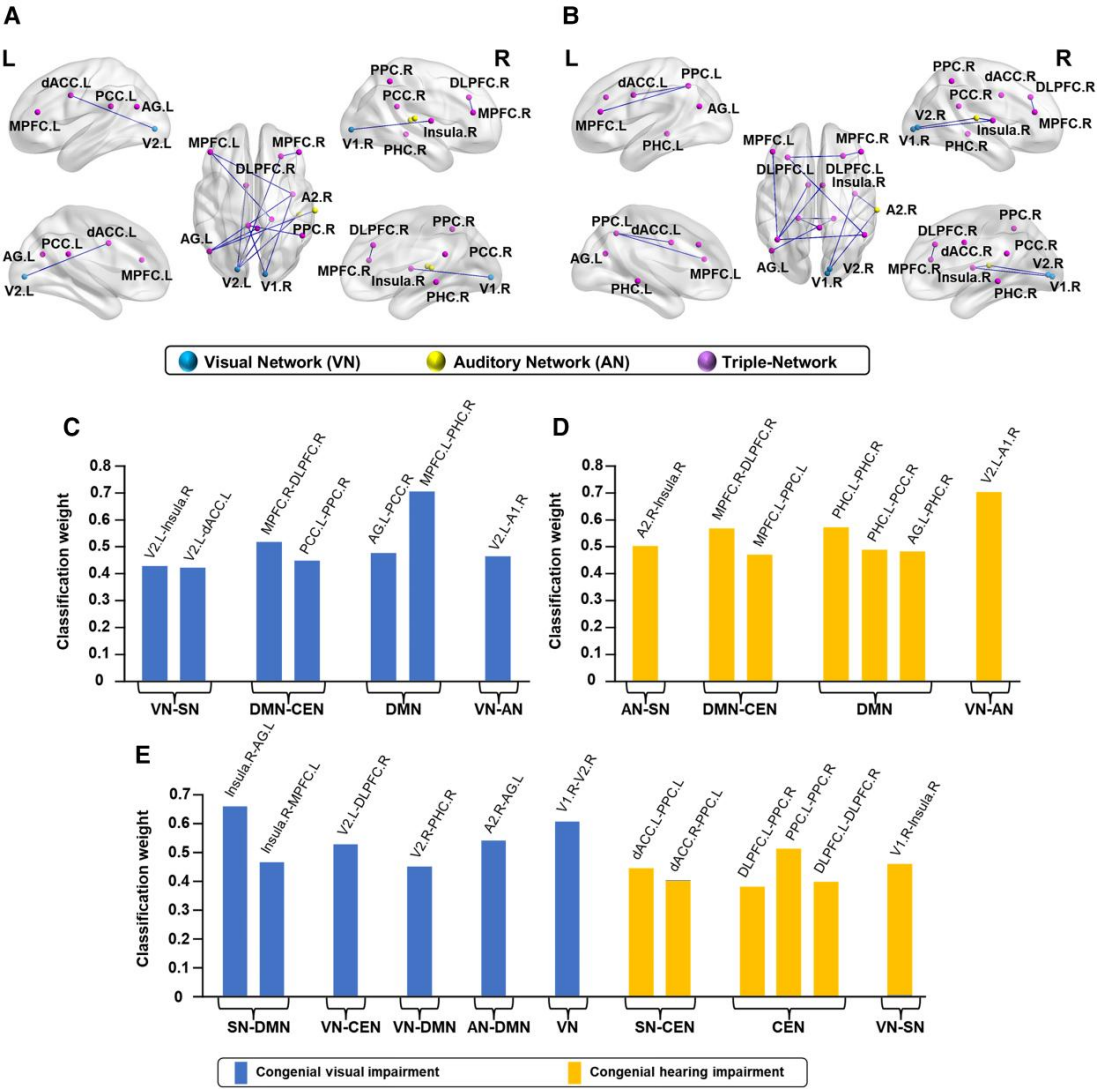
**Functional connectivity map of the top 5% of classification weights in the SVM model based on functional connectivity coefficient features.** To investigate the commonalities and differences in brain network remodeling between congenital visual impairments and congenital hearing impairments, we constructed a binary classification model against a normal group using Support Vector Machine (SVM) and then extracted the features ranked in the top 5% of weights from the resultant model. (**A**) Compared with normal group, the functional connectivity map of the top 5% of features with the highest classification significance in the SVM model for children with congenital visual impairments. (**B**) Compared with normal group, the functional connectivity map of the top 5% of features with the highest classification significance in the SVM model for children with congenital hearing impairments. (**C** and **D**) Comparison of the model results of **A** and **B** shows the similar or common functional connectivity patterns between brain regions in children with congenital visual impairments **C** and children with congenital auditory impairments **D**. (**E**) Comparison of the model results of **A** and **B** shows the different functional connectivity patterns between brain regions in children with congenital visual impairments and children with congenital hearing impairments. Blue dots represent the visual network, yellow dots represent the auditory network and purple dots denote the triple-network (including the default mode network, salience network and central executive network). Blue lines indicate statistically significant reductions in functional connectivity compared with the normal group. L and R respectively represent the left and right.

In the alpha frequency band, where both SVM models exhibited the highest classification accuracy, we extracted and compared the top 5% of ranked functional connections weights (5% of all connections, i.e. 13/276). Our results found that the changes in functional connectivity in children with congenital visual and hearing impairments, manifested by abnormalities between the SN or AN and SN, between DMN and CEN, between the VN and AN and within the DMN. These core nodes of the network system mainly include the left V2, right A1/A2, bilateral MPFC, bilateral PHC, bilateral PCC, left AG, left dACC, right INS and right PPC. The specific connections between specific brain regions can see Fig. 3C and D. In addition, different functional connectivity changes were observed between congenital visual impairment and congenital hearing impairment in the model compared to the normal group, such as abnormalities in the SN and DMN in children with congenital visual impairment and abnormalities in the SN and CEN in children with congenital hearing impairment. The specific connections between specific brain regions can be seen in Fig. 3E.

### The spatial distribution of brain rhythm disturbance

To further investigate the central remodeling mechanism of congenital visual and hearing impairments, we compared and analysed the cross-frequency coupling coefficients (i.e. mutual information values, MI values) of 24 ROIs [independent sample *t*-test, the significance level alpha is <0.05, corrected by false discovery rate correction].

**Figure 4:**
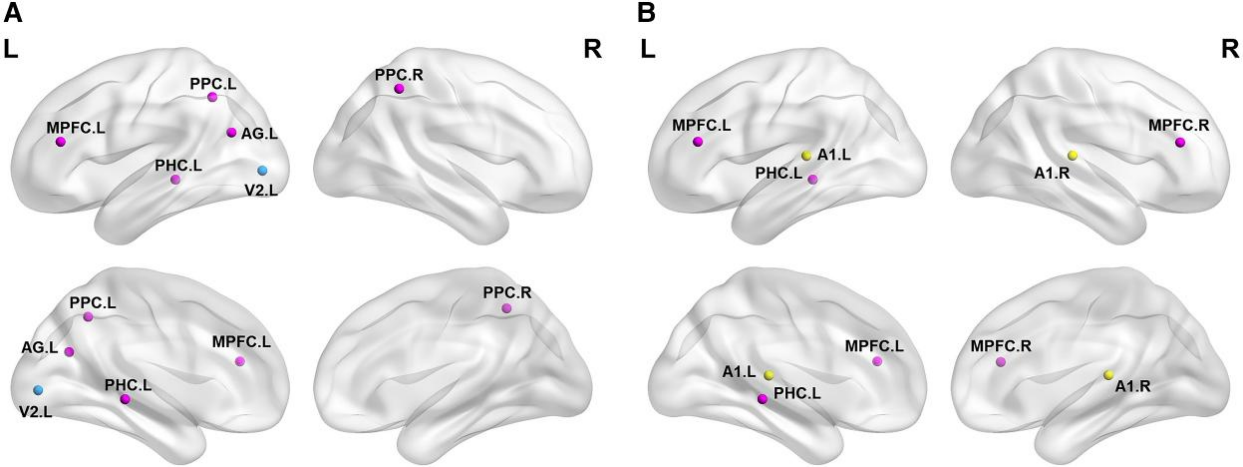
**Differences in the strength of cross-frequency coupling in the cortex of individuals with congenital sensory impairments compared to the normal group.** To further investigate the mechanisms of central remodeling in congenital visual and hearing impairments, we compared and analysed the cross-frequency coupling coefficients (i.e. mutual information values, MI values) of 24 regions of interest (ROIs) using an independent sample *t*-test, where the significance level alpha is <0.05, corrected by false discovery rate (FDR) correction. (**A**) Compared with the normal group, children with congenital visual impairments showed enhanced theta–gamma coupling in the left V2, PHC, AG and MPFC (*t* > 3.13, df = 80, *P* < 0.05); in addition, the theta–gamma coupling in the bilateral PCC was weakened (*t* < −3.46, df = 80, *P* < 0.05). (**B**) Compared with the normal group, children with congenital hearing impairments showed enhanced theta-gamma coupling in the bilateral A1, left PHC and MPFC (*t* > 3.45, df = 80, *P* < 0.05). Blue dots represent the visual network, yellow dots represent the auditory network and purple dots denote the triple-network (including the default mode network, salience network and central executive network). L, left; R, right.

The results showed that, compared with the normal group, enhanced theta–gamma coupling in children with congenital visual impairment was mainly concentrated in core area of processing advanced visual information, such as the left V2 brain region (*t* > 3.13, df = 80, *P* < 0.05); whereas, in children with congenital hearing impairment, the brain area of enhanced theta–gamma coupling was located in core regions processing advanced auditory information, such as the bilateral A1 (*t* > 3.45, df = 80, *P* < 0.05). On the other hand, not limited to the sensory cortex, children with either congenital visual or hearing impairment showed enhanced theta–gamma coupling in non-visual or auditory cortical areas such as the left PHC and left MPFC, exhibiting similar spatial distribution characteristics. Furthermore, children with congenital visual impairment showed weakened theta–gamma coupling in the bilateral PPC (*t* < −3.46, df = 80, *P* < 0.05). The specific brain regions can be seen in Fig. 4.

In other brain regions, no significant theta–gamma coupling abnormalities were observed in children with congenital visual/auditory impairments.

## Discussion

**Figure 5:**
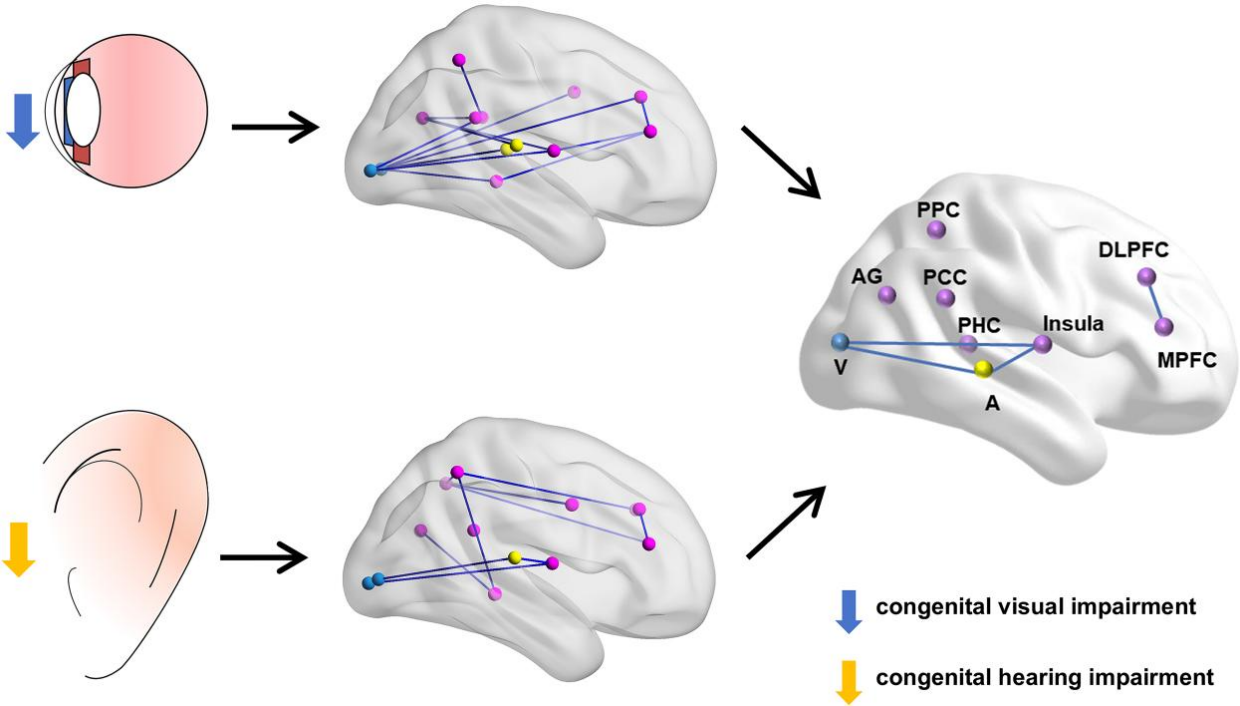
**Schematic summary of common brain network changes and affected brain regions in congenital visual and hearing impairments.** Blue line: The weakened functional connectivity in congenital sensory impairments compared to normal children (middle); shared functional connectivity between congenital visual and hearing impairments (right). Blue dots represent the visual network, yellow dots represent the auditory network and purple dots denote the triple-network (including the default mode network, salience network and central executive network). Blue downward arrow represents the congenital visual impairment group; yellow downward arrow represents the congenital auditory impairment group.

The aim of this study is to investigate whether different sensory deficits, specifically congenital visual/hearing impairments, share common or similar disruptions in sensory input transmission pathways and overlapping patterns of neural network reorganization. We employed sophisticated EEG analysis techniques and utilized a purely data-driven approach, incorporating SVM classification, to select and rank brain network functional connections. Our findings clearly indicate that children with congenital visual/hearing impairments exhibit distinct and spatially distributed patterns of attenuation and dysrhythmia in specific brain regions at the central level. Moreover, there is widespread weakening of brain network connections, presenting characteristics of a triple-network abnormalities. Within these central reorganization characteristics, there are similar functional connections patterns shared between the two sensory deficits. These common characteristics include connections between the sensory cortex and the insula, connections between the dorsolateral prefrontal cortex and the medial prefrontal cortex, connections between the audio-visual cortex, and other core brain regions of the triple-network, as illustrated in Fig. 5. These findings advance our understanding of the central mechanisms underlying sensory deficits induced by congenital visual/hearing impairments and their impact on higher cognitive functions.

### Cortical adaptations to decreased bottom-up sensory input

The results of source localization analysis indicate that children with congenital visual impairments show a significant reduction in power in the occipital cortex. Similarly, decreased cortical activity was observed in the temporal cortex in children with congenital hearing impairments. These observations are primary features of congenital sensory deficits within the cerebral cortex and are consistent with the general deprivation hypothesis. Specifically, functional defects in peripheral sensory channels lead to a reduction in the transmission of visual/auditory information. This situation can be seen as an adaptive change in higher sensory cortical areas caused by the disruption in the bottom-up neural transmission pathways. Moreover, another significant commonality is the observed reduction in cortical activity in the alpha frequency band, which has been demonstrated to be influenced by sensory processing and attention regulation.^35^ The alpha frequency band acts as an inhibitor, helping to suppress irrelevant stimuli.^36^ For children with congenital vision or hearing loss, due to the reduced sensory input from the eyes or ears, the brain receives significantly fewer visual or auditory signals. This implies that the amount of information the brain needs to process in these sensory channels also decreases. With fewer processing tasks, the brain no longer needs to utilize alpha waves to suppress a large amount of irrelevant information. However, the development of the alpha frequency band is experience-dependent,^37^ which may highlight the importance of early vision/hearing correction.

Additionally, the activation of the occipital cortex observed in children with congenital hearing impairment can be interpreted as a compensatory response within the central nervous system, consistent with findings from previous studies. Essentially, functional defects in sensory channels lead to a reduced capacity to receive and process external stimuli, triggering regulatory mechanisms within the central nervous system. The brain attempts to compensate for these defects by reallocating resources and adjusting neural connections. This compensatory mechanism is a crucial component of the brain's plasticity, where central compensation refers to undamaged neural regions in the central nervous system compensating for the function of damaged areas through adaptive changes. These compensatory changes are not limited to the regeneration and replacement of neurons, synaptic remodeling, and usually involve functional reorganization.^38^ That is, when a sensory channel cannot function normally, the brain may reorganize neural circuits, redistributing functionalities originally associated with that channel to other channels or brain regions to complete tasks. This reorganization helps individuals continue to receive and process information from other sensory channels, adapting to various needs in daily life. For example, the activation of the occipital cortex (usually primarily responsible for visual processing) in children with hearing loss may indicate that the visual system is, to some extent, taking on compensatory functional processing for auditory inputs.^39^ Moreover, it is noteworthy that the activation of the occipital cortex in children with congenital hearing impairment occurs in the beta frequency band. Beta frequency band (approximately 13–30 Hz) brain waves are generally associated with active thinking and information processing states.^40^ They often occur in focused attention, perceptual processing and cognitive tasks and are considered to play a particularly important role in maintaining current sensory-motor or cognitive states.^41,42^ This means that, for children with hearing impairment, the visual system may need to undertake more information processing tasks and exhibit enhanced higher-level cognitive functions and focused attention, demonstrating higher activity levels in the beta frequency band.

### Common brain network reorganization patterns in visual /hearing impairments

Based on the SVM classification results and the sorting of weightings for functional connections, our research has revealed extensive abnormalities in brain network connections for children with congenital visual/hearing impairments. These abnormalities primarily involve brain regions related to emotions, attention, memory and other functions, including the insula, cingulate gyrus, parahippocampal gyrus and frontal cortex. Notably, the brain network changes resulting from visual and auditory sensory deficits exhibit similar alterations in neural pathways. Specifically, there are abnormal connections between the visual and auditory cortex and the right insula. In the developing human brain, the maturation of the insula pathway is crucial for supporting complex and flexible cognitive processes.^43^ Additionally, we observed common connectivity patterns in both groups, manifesting as abnormalities in within-network and between-network connections of the triple-network model. Based on the theoretical foundation of the triple-network model,^44^ we hypothesize that disruptions in the processing of sensory visual and auditory information may impact emotions and bodily functions by modulating the activity between the DMN and CEN.^45^ These results clearly indicate that congenital sensory deficits in vision and hearing lead to abnormalities in the triple-network model, resulting in abnormal interactions between multiple brain networks, especially those involved in sensory processing, emotional experience, and attention control.^46^ This suggests that congenital hearing and visual impairments share a similar disrupted sensory input transmission pathway and overlapping brain networks, exhibiting similar activation and connectivity patterns but affecting memory, emotional cognition and attention states through different spatial locations.

### Spatial distribution of brain rhythmic disturbances

Cross-frequency coupling plays a significant role in numerous cognitive activities and represents a potential physiological mechanism for the brain to transmit and store information, encoding strategies for inter-regional information exchange.^47^ The cross-frequency coupling analysis revealed that congenital visual /hearing impairments exhibit abnormal phase-amplitude coupling in multiple brain regions. These abnormalities demonstrate both shared and specific spatial disturbances characteristics. That is, visual impairment induces rhythmic disturbances in the visual cortex, while auditory impairment induces rhythmic disturbances in the auditory cortex. Furthermore, disturbances in regions such as the PHC and frontal cortex are observed in both groups. This finding provides new insights into the neural reorganization mechanisms of sensory deficits. Regardless of the type of impaired sensory input, power reduction occurs in the sensory cortex, and theta–gamma coupling is enhanced. The abnormal connectivity of brain networks shows characteristics of a triple-network disturbance. It is particularly important to note the abnormal dysregulation in the PHC and frontal brain regions, suggesting that congenital visual/hearing impairments may lead to anomalies in working memory, executive function and emotional control.^48^ Therefore, in future research, it is essential to focus on the development of attention, working memory and other aspects. This study holds significant importance for a deeper understanding of central cortical rhythm disruptions caused by visual and auditory sensory deficits and provides potential guidance for improving the perception and cognitive functions of affected individuals. Our findings will provide substantial support for future clinical interventions and rehabilitation strategies.

### Limitations

This study is a cross-sectional study with a limited sample size. Therefore, longitudinal observations such as follow-up studies are needed to provide deeper insights into the central reorganization mechanisms triggered by visual and auditory sensory deficits. Additionally, due to the limitations of spatial resolution in electroencephalography (EEG), it is possible that some significant results may not have been detected. Future research should consider integrating functional magnetic resonance imaging technology to complement the information on brain region functionality and network connectivity changes in high-resolution spatial settings. Furthermore, the changes in higher cognitive functions need to be further validated through methods such as psychological experiments and scale assessments, and the expression of central functional reorganization after clinical interventions should be evaluated. Innovative data mining algorithms can also be employed in future studies to deepen the significance of classification and prediction.

## Conclusion

Congenital visual/hearing impairments lead to central reorganization with common features in brain function. The primary characteristics of central reorganization due to sensory channel deficits is the adaptive changes in the primary sensory cortices, specifically the reduction in alpha frequency band power in the occipital and temporal cortices. Concurrently, compensatory changes in the cerebral cortex occur, involving the reallocation of brain resources and adjustments in neural connections to compensate for channel deficits. Common characteristics of brain network disruptions in visual /hearing impairments include disturbances in the triple network, affecting rhythmic patterns and functional connections in multiple brain regions, particularly those associated with sensory perception, emotions and cognition. These findings are of significant importance for understanding the central cortical rhythm disruptions caused by sensory deficits, providing potential guidance for improving the perception and cognitive functions of affected individuals. They offer substantial support for future clinical interventions and rehabilitation strategies.

## Data Availability

The raw data and analysis scripts/codes for this study are available upon request from the corresponding author, subject to relevant data use conditions. All personal data processing complies with data protection regulations.
